# A comparative study on the efficacy of artesunate plus sulphadoxine/pyrimethamine versus artemether-lumefantrine in eastern Sudan

**DOI:** 10.1186/1475-2875-6-92

**Published:** 2007-07-15

**Authors:** Ebtihal A Mukhtar, Nahla B Gadalla, Salah-Eldin G El-zaki, Izdihar Mukhtar, Fathi A Mansour, Ahmed Babiker, Badria B El-Sayed

**Affiliations:** 1 Tropical Medicine Research Institute, P.O. Box 1304, Khartoum, Sudan

## Abstract

**Background:**

A combination of artesunate (AS) plus sulphadoxine/pyrimethamine (SP) as first-line and artemether-lumefantrine (AL) as second-line treatment are currently recommended against uncomplicated *P. falciparum *infection in Sudan. However, there is limited information on the efficacy of ACTs in the country and only one report of PCR-corrected results for AS/SP only.

**Methods:**

The WHO protocol for the assessment of antimalarial drug efficacy for the treatment of uncomplicated falciparum malaria was employed. Artesunate plus sulphadoxine/pyrimethamine (AS/SP) was compared to artemether-lumefantrine (AL) in a 28-day follow up. Samples that were classified as early treatment failure (ETF), late treatment failure (LCF) or late parasitological failure (LPF) were genotyped for *msp-1 *and *msp*-*2 *genes to differentiate recrudescence from reinfection.

**Results:**

A total of 178 patients were screened and 160 met the enrolment criteria and were recruited to the study of which 157 (98.1%) completed the follow up and had an analysed treatment outcome. On the AS/SP arm, three (0.038%) patients were lost during the follow-up, two on day 1 and one on day 7, and 77 (96.3) completed the study, while all 80 (100%) patients completed the follow up in the AL arm. In the per protocol analysis for AS/SP the treatment outcome for patients who completed the follow-up were as follows: adequate clinical and parasitological response (ACPR); 84.4% ETF; 1.3%, LCF; 3.9%, (LPF); 10.4%. For the AL arm the out come was as follows, ACPR; 90%, ETF; 0%, LCF; 6.3% and LPF; 3.8%. However, when PCR-corrected, 6.5% (5/77) of patients treated with AS/SP maintained parasites from their primary infection, while (7/80) in the AL group maintained their initial parasite genotype. Therefore, PCR-corrected efficacy was 93.5% in the AS/SP treated group and for AL it was 91.3%.

**Conclusion:**

Both AS/SP and AL are highly effective for the treatment of uncomplicated falciparum malaria in eastern Sudan. However, AS/SP appears to have a slightly higher efficacy than AL, this may be due to patient compliance with the repeated dose rather than drug efficacy.

## Background

Malaria remains one of the major public health problems in Africa. It is estimated that 7.5 to 10 million cases of malaria occur in Sudan every year [1]. Control mainly depends on treating symptomatic cases with available chemotherapeutics. The WHO recommends artemisinin- based combination therapies (ACT) for treatment of uncomplicated malaria. Due to decreased sensitivity of *Plasmodium falciparum *to chloroquine [2] and sulphadoxine/pyrimethamine (SP) [3,4], the National Malaria Control Programme (NMCP) of Sudan has recently adopted ACT for treatment of uncomplicated malaria [5]. Artemisinins are known to have a marked effect on reducing parasitaemia even when multi-drug resistant parasites are present. However, their short half-life warrants that a second drug with long half-life is administered concurrently.

The current treatment protocol for uncomplicated falciparum malaria in Sudan is artesunate plus sulphadoxine/pyrimethamine, as first-line and artemether-lumefantrine as second-line of treatment [5]. There have been few studies on the efficacy of AS/SP [6-9] and only one published report on the efficacy of AL in Sudan [8]. Although these studies have reported high efficacy to both combinations, ranging from 99.8% to 100%, they did not show whether the small proportion of treatment failures was due to a recrudescence of existing parasites or acquisition of a new infection, except for one recent study which reported the PCR-corrected efficacy of AS/SP [9]. It was, therefore, decided to closely monitor patients for 28 days after treatment with AS/SP or AL, and the results were genotyped to indicate recrudescence versus new infections.

## Methods

### Sample size

A trial with 80 subjects in each arm would have at least 80% power (using a significance level of 0.10) to detect a difference between the two combinations if the failure rate detected after treatment is 20% in the AS/SP arm (based on the reported failure of SP of approximately 15 – 20% in Sudan) and 5% in AL arm, allowing for 10% loss to follow-up.

### Study population and field survey

The study was carried out in three villages in eastern Sudan, southwest of Gedarif town (Abunaja Juama, Abunaja Bargo and Asar). A community-based survey was conducted during the transmission seasons (October to December) of 2004 and 2005 to identify symptomatic patients that were microscopically diagnosed with mono infection of *P. falciparum*. Patients that satisfied the enrolment criteria according to the WHO protocol for the assessment and monitoring of antimalarial drug efficacy for the treatment of uncomplicated falciparum malaria [10] were recruited after they provided written informed consent to participate. However, due to the low endemicity of malaria in this area all age groups were recruited. Patients were randomly allocated to receive either AS/SP or AL by a simple random technique of a hat draw. Pregnant women were not enrolled as per the national protocol for uncomplicated malaria recommendations.

### Sample collection

Finger prick blood samples were collected and thick and thin smears were prepared on a patient's first attendance. When the patient was enrolled to the study, a finger prick sample was collected on days 0, 1, 2, 3, 7, 14, 21, and 28 on a glass slide and a parallel drop of blood was collected on filter paper. The filter paper was air-dried and stored in self-sealing plastic bags for molecular analysis.

### Microscopic diagnosis

Thick and thin blood smears were stained with Giemsas' stain for ten minutes. The slides were read by two independent experienced microscopists. Parasite count was performed by counting asexual parasites against 200 white blood cells (WBC), and parasite density calculated assuming an average of 6,000 WBC in the population. Ten percent of the slides were read by a third microscopist in Khartoum for quality control. In case of disagreement the majority result was taken.

### Treatment and follow up schedule

Each patient was allocated randomly to either AS/SP or AL, treatment was administered under supervision for the AS/SP group, while only one dose of the AL was supervised and the second dose was given to the patient for self-administration. Patients were asked to return to the clinic on days 1, 2, 3, 7, 14, 21, and 28 or whenever they did not feel well. When a patient failed to come to the clinic on any scheduled day, a member of the team visited the home, gave them the dose and obtained the sample unless the patient was not found or refused to give the sample. Those who refused to give their sample (withdrawn) or missed more than one day of follow-up were excluded from the analysis.

Patients, who were positive by microscopy and refused to participate in the study or did not meet the enrolment criteria, were provided with AS/SP for self-administration whereas pregnant women were referred to Gedarif Teaching Hospital as recommended by the national protocol.

### In vivo analysis

A per protocol analysis was performed employing the WHO excel sheet [11].

Samples that were positive by microscopy on any of the follow-up days were classified as early treatment failure (ETF), late parasitological failure (LPF), or late clinical failure (LCF). Adequate clinical and parasitological response (ACPR) was defined as complete absence of microscopically detectable parasitaemia during the follow-up period, according to the protocol [10].

### Molecular analysis

Samples that were classified as ETF, LCF or LPF were genotyped by PCR as previously described for *msp-1 *[12] and *msp*-2 [13,14] on day 0, 1, 2, 3 and failure (day 7 to 28) to differentiate recrudescence from reinfection. Unpublished observations indicate the necessity of day 0 to day 3 sampling to accurately identify recrudescence from re-infection.

### Ethical considerations

The study was reviewed and ethical clearance was obtained from the National Ethical Committee, Federal Ministry of Health, Sudan. Individual written informed consent was obtained from all participants or their legal guardians.

## Results

### In vivo outcome

178 patients were screened and 160 patients were enrolled and 157 completed the follow-up and had an analysable outcome. Of these, 77 patients were in the AS/SP arm and 80 were in the AL arm. Table 1 shows the description of the study population that successfully completed the follow-up.

**Table 1:** Description of study population receiving AS/SP or AL treatment

		Drug	
		AS/SP	AL

Age_years	Mean (95% CI)	17.88 (14.48 – 21.28)	18.45 (14.84 – 22.06)
Temperature Day 0	Mean (95% CI)	37.46 (37.46 – 37.70)	37.59 (37.35 – 37.83)
Sex	Female n (%)	39 (48.8)	47(58.8)
	Male n (%)	41 (51.2)	33(41.2)

A total of 77 patients completed the follow-up on the AS/SP group and 84.4% had an adequate clinical and parasitological response (ACPR). Of the 80 patients that received AL and completed the follow-up, 90% had successfully cleared their parasitaemia by day 28. Table 2 summarizes the outcome of treatments in each group.

**Table 2:** *In vivo *outcome of AS/SP and AL

	Prevalence n (%)	
	AS/SP	AL
ETF	1 (1.3)	0 (0)
LCF	3 (3.9)	5 (6.3)
LPF	8 (10.4)	3 (3.8)
ACPR	65 (84.4)	72 (90)
Total analysed	77 (96.3)	80 (100)
WITH	3 (3.7)	0 (0)
TOTAL	80	80

ETF; early treatment failure; LCF: late clinical failure; LPF: late parasitological failure; ACPR: adequate clinical and parasitological response; WITH: withdrawal; as analysed by the WHO worksheet.

### Genotyping

Of the 157 patients diagnosed with *P. falciparum *on day 0 and who completed the follow up twenty patients had microscopically detectable parasitaemia on at least one day of the follow-up. Of these twelve received AS/SP and the remaining eight received AL. These samples were genotyped for *msp-1 *and *msp-2 *genes.

In the AS/SP group, five patients (41.7 %) showed different allelic forms and were classified as reinfection, while seven (58.3) retained the same allelic pattern and were classified as recrudescence of parasites. Only one (1.25%) patient showed a different genotype in the AL group and the remaining 7 (87.5%) retained their initial parasite genotype. Hence, PCR-corrected efficacy was 93.5% and 91.3% for AS/SP and AL, respectively.

## Discussion

Artemisinin derivatives are highly effective antimalarial compounds against different stages of *Plasmodium *development [15]. In order to prolong the efficacy of these drugs, it is recommended that they should only be used in combination with another effective drug that has a longer half-life. This current consensus of antimalarial therapy has led to the adoption of ACT in Sudan where chloroquine (CQ) has reached high levels of treatment failure [2,3,16].

In Sudan, the current recommendation for uncomplicated malaria are AS/SP and AL as first and second-line treatment, respectively [5] with 100% efficacy in Damazin, Kosti, Malakal and New Halfa [6-9,17] and 99.3% in Kassala [7].

In the present study, PCR-corrected results showed efficacy of AS/SP to be 93.5% in the Gedarif area. Although this is lower than the reports mentioned above, it is not surprising. Samples analysed in the present study were collected in 2004 and 2005, a time coinciding with increased use of SP mono-therapy due to the failure of CQ, which may have lead to increased levels of resistance to this drug. Although there is no recent report of SP clinical efficacy in our study area, alleles associated with SP failure have been reported to be increasing gradually. Triple mutants on *dhfr*-108Asn, *dhfr*-59 and *dhps*-540Leu have rapidly increased in this area between 1993 to 1999 [18]. Data from this area shows that these alleles have reached higher levels in 2003 and 2004 just before the implementation of ACTs [19].

The partial failure of AL (8.9%) may be explained by inabsorption of lumefantrine due to low fat diet a situation that may be common in rural areas. In addition, recrudescence of AL-treated individuals has been reported in studies from other parts of the world [20-22].

**Figure 1:**
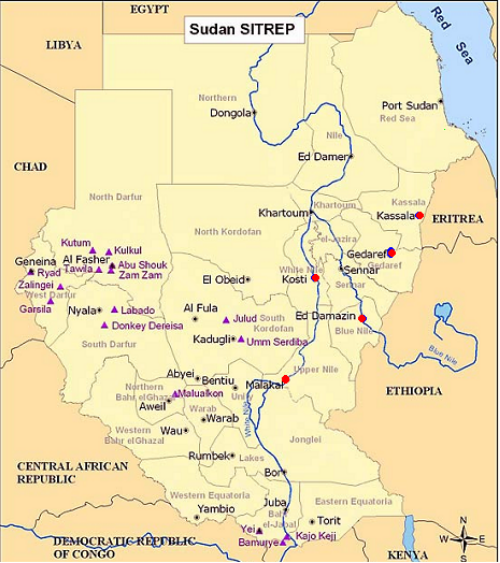
**Map of Sudan**. Red circles indicate the town locations reported in previous studies (Ed Damazin, Kosti, Kassala and Malakal) compared to this study (Gedaref). Other areas except Kassala are on the Nile River bank and have irrigated agricultural activities.

The present study has provided detailed PCR-corrected results of AS/SP and AL efficacy in eastern Sudan. Although the results may seem in disagreement with others, this is mainly due to the different transmission intensities in Sudan. In irrigated areas where there is perennial transmission such as Damazin, New Halfa, Malakal and Kosti (Figure 1) there is a higher chance of recombination within the parasite population. However, in areas of low seasonal transmission such as the Gedarif area, recombination may be much lower leading to the lower multiplicity of infection (MOI). This may have led to underestimation of reinfection in our study. In addition parasite tolerance to the lumefantrine component persisting at a low levels than required to kill parasites that have caused new infection may also contribute to high levels of failure [23].

## Conclusion

Artemisinin-based combination therapies are considered to be highly effective antimalarials available in endemic countries. Artemisinin is highly effective in eliminating parasitaemia, however the partner drug is an important component in prolonging the usefulness of this combination. The results shown here call for continuous monitoring of the efficacy of ACT in eastern Sudan.

## Authors' contributions

EM carried out the sample collection, molecular analysis, data entry and contributed to drafting the manuscript, NG conceived of the study, contributed to its design, sample collection, data analyses and drafted the manuscript, SE contributed to the sample collection, microscopy and molecular analysis. IM and FM contributed to the sample collection and molecular analysis. AB participated in study design, data management and analyses. BE conceived of the study, participated in its design, analyses and helped to draft the manuscript. All authors read and approved the final manuscript.

## References

[B1] Malik EM (2004). Malaria in Sudan: past, present and the future. Gezira Journal of Health Sciences.

[B2] Babiker HA, Pringle SJ, Abdel-Muhsin A, Mackinnon M, Hunt P, Walliker D (2001). High-level chloroquine resistance in Sudanese isolates of Plasmodium falciparum is associated with mutations in the chloroquine resistance transporter gene pfcrt and the multidrug resistance Gene pfmdr1. J Infect Dis.

[B3] Adam I EMI ( 2005). Resistance of P. falciparum to antimalarial drugs in Sudan. Khartoum Med J.

[B4] Salah MT, Mohammed MM, Himeidan YE, Malik EM, Elbashir MI, Adam I (2005). A randomized comparison of sulphadoxine-pyrimethamine and combination of sulphadoxine pyrimethamine with chloroquine in the treatment of uncomplicated falciparum malaria in Eastern Sudan. Saudi Med J.

[B5] Malik EM, Mohamed TA, Elmardi KA, Mowien RM, Elhassan AH, Elamin SB, Mannan AA, Ahmed ES (2006). From chloroquine to artemisinin-based combination therapy: the Sudanese experience. Malar J.

[B6] Adam I, Ali DM, Abdalla MA (2006). Artesunate plus sulfadoxine-pyrimethamine in the treatment of uncomplicated Plasmodium falciparum malaria during pregnancy in eastern Sudan. Trans R Soc Trop Med Hyg.

[B7] Elamin SB, Malik EM, Abdelgadir T, Khamiss AH, Mohammed MM, Ahmed ES, Adam I (2005). Artesunate plus sulfadoxine-pyrimethamine for treatment of uncomplicated Plasmodium falciparum malaria in Sudan. Malar J.

[B8] Mohamed AO, Eltaib EH, Ahmed OA, Elamin SB, Malik EM (2006). The efficacies of artesunate-sulfadoxine-pyrimethamine and artemether-lumefantrine in the treatment of uncomplicated, Plasmodium falciparum malaria, in an area of low transmission in central Sudan. Ann Trop Med Parasitol.

[B9] Ibrahium AM, Kheir MM, Osman ME, Khalil IF, Alifrangis M, Elmardi KA, Malik EM, Adam I (2007). Efficacies of artesunate plus either sulfadoxinepyrimethamine or amodiaquine, for the treatment of uncomplicated, Plasmodium falciparum malaria in eastern Sudan. Annals of Tropical Medicine and Parasitology.

[B10] WHO (2003). Assessment and monitoring of antimalarial drug efficacy for the treatment of uncomplicated malaria.

[B11] WHO Excel sheet for Resistance Data Collection and analysis. http://www.who.int/malaria/rbm/Attachment/20041108/resistance_datacollection.xls.

[B12] Cavanagh DR, Elhassan IM, Roper C, Robinson VJ, Giha H, Holder AA, Hviid L, Theander TG, Arnot DE, McBride JS (1998). A Longitudinal Study of Type-Specific Antibody Responses to Plasmodium falciparum Merozoite Surface Protein-1 in an Area of Unstable Malaria in Sudan. J Immunol.

[B13] Ntoumi F, Contamin H, Rogier C, Bonnefoy S, Trape JF, Mercereau-Puijalon O (1995). Age-dependent carriage of multiple Plasmodium falciparum merozoite surface antigen-2 alleles in asymptomatic malaria infections. Am J Trop Med Hyg.

[B14] Zwetyenga J, Rogier C, Tall A, Fontenille D, Snounou G, Trape JF, Mercereau-Puijalon O (1998). No influence of age on infection complexity and allelic distribution in Plasmodium falciparum infections in Ndiop, a Senegalese village with seasonal, mesoendemic malaria. Am J Trop Med Hyg.

[B15] Terkuile F, White NJ, Holloway P, Pasvol G, Krishna S (1993). Plasmodium falciparum: In Vitro Studies of the Pharmacodynamic Properties of Drugs Used for the Treatment of Severe Malaria. Experimental Parasitology.

[B16] Tagelsir N, Ibrahim Z, Medani A, Salih O, Hamad A, Giha H, El-Agib A, Khan B, Saeed N, Ibrahim M (2006). High frequency of Plasmodium falciparum PfCRT K76T and PfpghN86Y in patients clearing infection after chloroquine treatment in the Sudan. Acta Trop.

[B17] Adam I, IE AE, Idris SM, Malik EM, Elbashir MI (2005). A comparison of the efficacy of artesunate plus sulfadoxine-pyrimethamine with that of sulfadoxine-pyrimethamine alone, in the treatment of uncomplicated, Plasmodium falciparum malaria in eastern Sudan. Ann Trop Med Parasitol.

[B18] Abdel-Muhsin AM, Mackinnon MJ, Ali E, Nassir el KA, Suleiman S, Ahmed S, Walliker D, Babiker HA (2004). Evolution of drug-resistance genes in Plasmodium falciparum in an area of seasonal malaria transmission in Eastern Sudan. J Infect Dis.

[B19] Gadalla N (2006). The Impact of clearing asymptomatic Plasmodium falciparum parasitaemia on drug resistance genes in an area of seasonal transmission in Sudan. International Conference for Parasitology, ICOPA.

[B20] Dokomajilar C, Nsobya SL, Greenhouse B, Rosenthal PJ, Dorsey G (2006). Selection of Plasmodium falciparum pfmdr1 Alleles following Therapy with Artemether-Lumefantrine in an Area of Uganda where Malaria Is Highly Endemic. Antimicrob Agents Chemother.

[B21] Humphreys GS, Merinopoulos I, Ahmed J, Whitty CJ, Mutabingwa TK, Sutherland CJ, Hallett RL (2007). Amodiaquine and artemether-lumefantrine select distinct alleles of the Plasmodium falciparum mdr1 gene in Tanzanian children treated for uncomplicated malaria. Antimicrob Agents Chemother.

[B22] Sisowath C, Stromberg J, Martensson A, Msellem M, Obondo C, Bjorkman A, Gil JP (2005). In vivo selection of Plasmodium falciparum pfmdr1 86N coding alleles by artemether-lumefantrine (Coartem). J Infect Dis.

[B23] Hastings IM, Watkins WM (2006). Tolerance is the key to understanding antimalarial drug resistance. Trends in Parasitology.

